# Improving the prognostic value of multimorbidity through the integration of selected biomarkers to the comprehensive geriatric assessment: An observational retrospective monocentric study

**DOI:** 10.3389/fmed.2022.999767

**Published:** 2022-10-31

**Authors:** Francesco Piacenza, Mirko Di Rosa, Massimiliano Fedecostante, Fabiana Madotto, Alberto Montesanto, Andrea Corsonello, Antonio Cherubini, Mauro Provinciali, Luca Soraci, Rosamaria Lisa, Silvia Bustacchini, Anna Rita Bonfigli, Fabrizia Lattanzio

**Affiliations:** ^1^Unit of Advanced Technology of Aging Research, Istituto di Ricovero e Cura a Carattere Scientifico (IRCCS) Istituto Nazionale di Ricovero e Cura per Anziani (INRCA), Ancona, Italy; ^2^Unit of Geriatric Pharmacoepidemiology and Biostatistics, Istituto di Ricovero e Cura a Carattere Scientifico (IRCCS) Istituto Nazionale di Ricovero e Cura per Anziani (INRCA), Cosenza, Italy; ^3^Geriatria, Accettazione geriatrica e Centro di ricerca per l'invecchiamento, Istituto di Ricovero e Cura a Carattere Scientifico (IRCCS) Istituto Nazionale di Ricovero e Cura per Anziani (INRCA), Ancona, Italy; ^4^Value-Based Healthcare Unit, Istituto di Ricovero e Cura a Carattere Scientifico (IRCCS) MultiMedica, Milan, Italy; ^5^Department of Biology, Ecology and Earth Sciences, University of Calabria, Cosenza, Italy; ^6^Scientific Direction, Istituto di Ricovero e Cura a Carattere Scientifico (IRCCS) Istituto Nazionale di Ricovero e Cura per Anziani (INRCA), Ancona, Italy

**Keywords:** multimorbidity (MM), functional impairment, death, hospitalization, epigenetic clock, protein biomarkers

## Abstract

**Background:**

Multimorbidity (MM) burdens individuals and healthcare systems, since it increases polypharmacy, dependency, hospital admissions, healthcare costs, and mortality. Several attempts have been made to determine an operational definition of MM and to quantify its severity. However, the lack of knowledge regarding its pathophysiology prevented the estimation of its severity in terms of outcomes. Polypharmacy and functional impairment are associated with MM. However, it is unclear how inappropriate drug decision-making could affect both conditions. In this context, promising circulating biomarkers and DNA methylation tools have been proposed as potential mortality predictors for multiple age-related diseases. We hypothesize that a comprehensive characterization of patients with MM that includes the measure of epigenetic and selected circulating biomarkers in the medical history, in addition to the functional capacity, could improve the prognosis of their long-term mortality.

**Methods:**

This monocentric retrospective observational study was conducted as part of a project funded by the Italian Ministry of Health titled “imProving the pROgnostic value of MultimOrbidity through the inTegration of selected biomarkErs to the comprehensive geRiatric Assessment (PROMOTERA).” This study will examine the methylation levels of thousands of CpG sites and the levels of selected circulating biomarkers in the blood and plasma samples of older hospitalized patients with MM (*n* = 1,070, age ≥ 65 years) recruited by the Reportage Project between 2011 and 2019. Multiple statistical approaches will be utilized to integrate newly measured biomarkers into clinical, demographic, and functional data, thus improving the prediction of mortality for up to 10 years.

**Discussion:**

This study's results are expected to: (i) identify the clinical, biological, demographic, and functional factors associated with distinct patterns of MM; (ii) improve the prognostic accuracy of MM patterns in relation to death, hospitalization-related outcomes, and onset of new comorbidities; (iii) define the epigenetic signatures of MM; (iv) construct multidimensional algorithms to predict negative health outcomes in both the overall population and specific disease and functional patterns; and (v) expand our understanding of the mechanisms underlying the pathophysiology of MM.

## Introduction

The number of people with multiple diseases, i.e., multimorbidity (MM), is expected to increase as a result of aging populations and a rise in long-term ailments. MM burdens individuals (65% of adults aged 65–84 years and 82% of those aged 85+ years) and healthcare systems due to the fact that it increases polypharmacy, hospital admissions, healthcare costs, dependency, and institutionalization (1–5). The association between MM and mortality is being investigated as certain studies have revealed a higher risk of death among older adults with MM compared to those without diseases (6, 7), although other studies found no differences (8, 9). The UK Academy of Medical Sciences has deemed it a research priority to reduce the burden of MM, thereby improving patients' quality of life and ensuring the healthcare system's sustainability (10).

One of the consequences of MM is the use of too many medications. This situation, known as polypharmacy, is typically defined as the concurrent intake of 5 or more drugs by a single individual. The prevalence of this condition in the older population grows with age and varies between 10 and 90%, depending on age group, definition used, healthcare, and the study's geographical setting (11, 12). In Italy, 30.3% of 2,057 participants (60% female; mean age 81.7 years) who visited a geriatric hospital emergency department were taking 6–9 drugs concomitantly, and 17.8% of the patients (13) presented with excessive polypharmacy (≥ 10 drugs). In a population of 319,185 Korean adults aged 65 and older, the prevalence of polypharmacy (≥ 6 medications) was estimated to be 86.4%, with 44.9% having excessive polypharmacy (≥ 11 medications) and 3.0% taking ≥ 21 medications (14). Polypharmacy increases the likelihood of adverse reactions, including drug-drug and drug-disease interactions (15), and is associated with a higher risk of inappropriate prescribing (12). However, it is unclear which unsuitable medication(s) could induce the onset of new comorbidities in older adults, consequently contributing to the severity of MM.

In contrast, substantial evidence has been presented on the relationship between MM and functional impairment (3, 16, 17). On the one hand, it has been found that the accumulation of chronic diseases may lead to functional impairment (16, 18). On the other, functional decline has been found to be associated with a higher risk of developing multiple chronic diseases, consequently increasing the severity and MM burden (3, 19). It has also been suggested that functional decline in older hospitalized patients may be a stronger predictor of poor outcomes than MM (3, 17, 20–22). Despite all the evidence, it is still unclear how these factors interact with one another and how polypharmacy may impact both.

Several studies have provided solid evidence that it is not merely by accident that chronic diseases may coexist (23–26). Both clinical experience and epidemiological research suggests that diseases often co-occur in an individual according to specific patterns (27, 28). However, no consensus has been reached on how these classifications can be applied to an operational definition to measure MM.

Several attempts have been made to develop a concise measure of MM and quantify its severity (29). However, a dearth of knowledge regarding its pathophysiology prevented the estimation of its severity in terms of outcomes. In order to bridge this gap, weighted disease measures, such as the Charlson Comorbidity Index (30), have been used to define the impact of multiple combinations of health/disease conditions on multiple outcomes, including mortality, quality of life, and resource utilization. However, the majority of studies aimed at estimating the risk of death in patients with MM by utilizing multiple comorbidity indexes indicated a high risk of bias (29).

In this context, a series of circulating physiological markers, including dehydroepiandrosterone sulfate (DHEAS), interleukin 6 (IL-6), C-reactive protein (CRP), cystatin C (Cyst-C), Alpha-2-macroglobulin (A2M), and soluble receptor for advanced glycation end-products (sRAGE), have been found to be associated with several age-related diseases and MM, indicating a potential clinical application for addressing patients with a higher risk of poor outcomes (31–34).

In addition to circulating biomarkers, DNA methylation patterns, which are associated with healthspan and the early prediction of death (35–38), may serve as powerful tools for elucidating the pathophysiology of MM and improving the estimation of its severity. Given the close association between DNA methylation, gene expression, and chromatin accessibility, a network analysis-based approach can be applied to identify several altered pathways that are enriched for disease-related changes (39).

The network medicine approach, which integrates big omics, imaging data, and clinical information, seeks to identify pathological interacting genes and proteins, revolutionizing disease knowledge and shifting the understanding of pathogenic phenomena from a reductionist to a holistic perspective (40).

Understanding the mechanisms of disease and comorbidity development, breaking them down into clusters, and disentangling the epigenetic and actionable components using a network medicine approach is of utter importance from a public health perspective. Currently, epigenetics combined with an innovative systems biology approach is useful for personalized coronary heart disease therapy (41). Furthermore, unlike DNA variants, epigenetic modifications are potentially pharmacologically reversible (42). This is why exploring DNA methylation patterns could: (i) highlight the altered mechanisms underlying MM and functional impairment; and (ii) identify treatments that may be implicated in the onset of new comorbidities or functional decline.

DNA methylation patterns include epigenetic clocks. These represent mathematical models that leverage the variability of a well-defined set of DNA methylation markers (CpG sites) to accurately predict all-cause mortality in later life (36). Multiple studies have demonstrated that epigenetic clocks can represent promising prognostic biomarkers for several diseases, including cardiovascular diseases (43), dementia (44), diabetes (45), and cancer (46), in addition to functional impairment (36) and frailty (47). However, no longitudinal studies have examined the alterations in DNA methylation status in relation to the onset of multiple comorbidities in older adults, with or without functional impairment, and their relationship with polypharmacy.

We hypothesize that a comprehensive characterization of patients with MM, by integrating the assessment of selected circulating and epigenetic biomarkers with the medical history, in addition to an assessment of functional capacity, could improve the long-term mortality prediction of these patients.

### Objectives

#### Primary objective

The main objective of this study is to evaluate the performance of the epigenetic clock expressed as *epigenetic age acceleration (epigenetic clock age–chronological age)* in predicting all-cause mortality in a cohort of MM older patients.

#### Secondary objectives

- The study of demographic, clinical, and functional variables in relation to mortality risk and rehospitalization rate after a 10-year follow-up.
- Evaluation of MM-cluster patient classification in relation to mortality risk and rehospitalization rate after a 10-year follow-up.
- The assessment of selected plasma proteins, including hsCRP, IL6, A2M, DHEAS, sRAGE, and Cys-C, in relation to clinical, biological, and functional outcomes.

Evaluation of *epigenetic age acceleration (epigenetic clock age–chronological age)* in relation to: (a) anthropometric parameters; (b) multiple patterns of MM; (c) disease distribution among patients; (d) functional status of the patients [Activities of Daily Living (48) and Cognitive Performance Scale (49) scores]; (e) hospitalization rate; (g) routine biological parameters; and (h) onset of new comorbidities related to the use of drugs in the context of polypharmacy.

## Materials and methods

### Study design

This monocentric retrospective observational study was conducted as part of a project funded by the Italian Ministry of Health (Grant No. GR-2019-12368606) titled “imProving the pROgnostic value of MultimOrbidity through the inTegration of selected biomarkErs to the comprehensive geRiatric Assessment (PROMOTERA).”

The PROMOTERA study aims to identify promising biomarkers of healthspan and mortality, such as selected circulating proteins and several DNA methylation biomarkers in the plasma and blood samples of hospitalized older patients recruited by the Reportage project (Trial registration number: NCT01397682; July 19, 2011). The Reportage project is (50) an ongoing prospective observational study involving older patients consecutively admitted to acute care wards in INRCA research hospitals located in the Italian cities of Ancona, Fermo, Cosenza, and Casatenovo. The project began in September 2011 with the aim of creating a large data repository on demographics, comprehensive geriatric assessments, clinical and diagnostic information, and molecular and biological data on older adults receiving acute care. This study included patients who were recruited until 2019. Potential participants were screened for eligibility after they were provided with information describing the study. Eligibility criteria included: age ≥ 65 years, admission to one of the INRCA research hospitals, and written informed consent. In the case of a participant's cognitive decline or impaired judgment, a proxy (relative or caregiver) was requested to give their additional consent. The absence of informed consent from the patient or caregiver (50) resulted in the participant's exclusion from the study. Baseline data were collected for each patient within the first 24 h of hospital admission. Baseline data were incorporated into a minimum data set comprising demographic information, multidimensional geriatric assessment data, clinical, biological, and diagnostic information routinely collected during hospitalization, also utilizing the interRAI Acute Care Instrument (51). Moreover, the Reportage dataset contained information on drug intake (prior to admission, during hospitalization, and at discharge) and diagnostic and treatment procedures performed during hospitalization (including information from X-rays, computerized tomography, ultrasounds, electrocardiograms, magnetic resonance imaging, etc.). The multidimensional geriatric assessment was repeated within 24 h prior to discharge. Blood samples were taken within 24 h of admission and stored at −80°C. A separate and detailed data sheet was used to collect data regarding *ad hoc* investigations to identify biological markers.

The PROMOTERA project aims to update follow-up information on the survival status of hospitalized Reportage Project patients for up to 10 years. It will also incorporate clinical, demographic, and functional data (already available from the Reportage project) with circulating and epigenetic biomarkers, thus improving the prognostic accuracy of patients with MM.

The Reportage project's protocol, recruitment, and informed consents have already been published (50) and approved by the Ethics Committee of the Italian National Institute of Health and Sciences on Aging (CE INRCA 20031, 04/02/2021). The approved PROMOTERA protocol includes DNA analyses on blood samples from the Reportage Project. Blood samples from the Reportage project are stored at −80° C in the biobank (BioGer) of the National Institute of Health and Sciences on Aging IRCCS-INRCA of Ancona.

**Figure 1:**
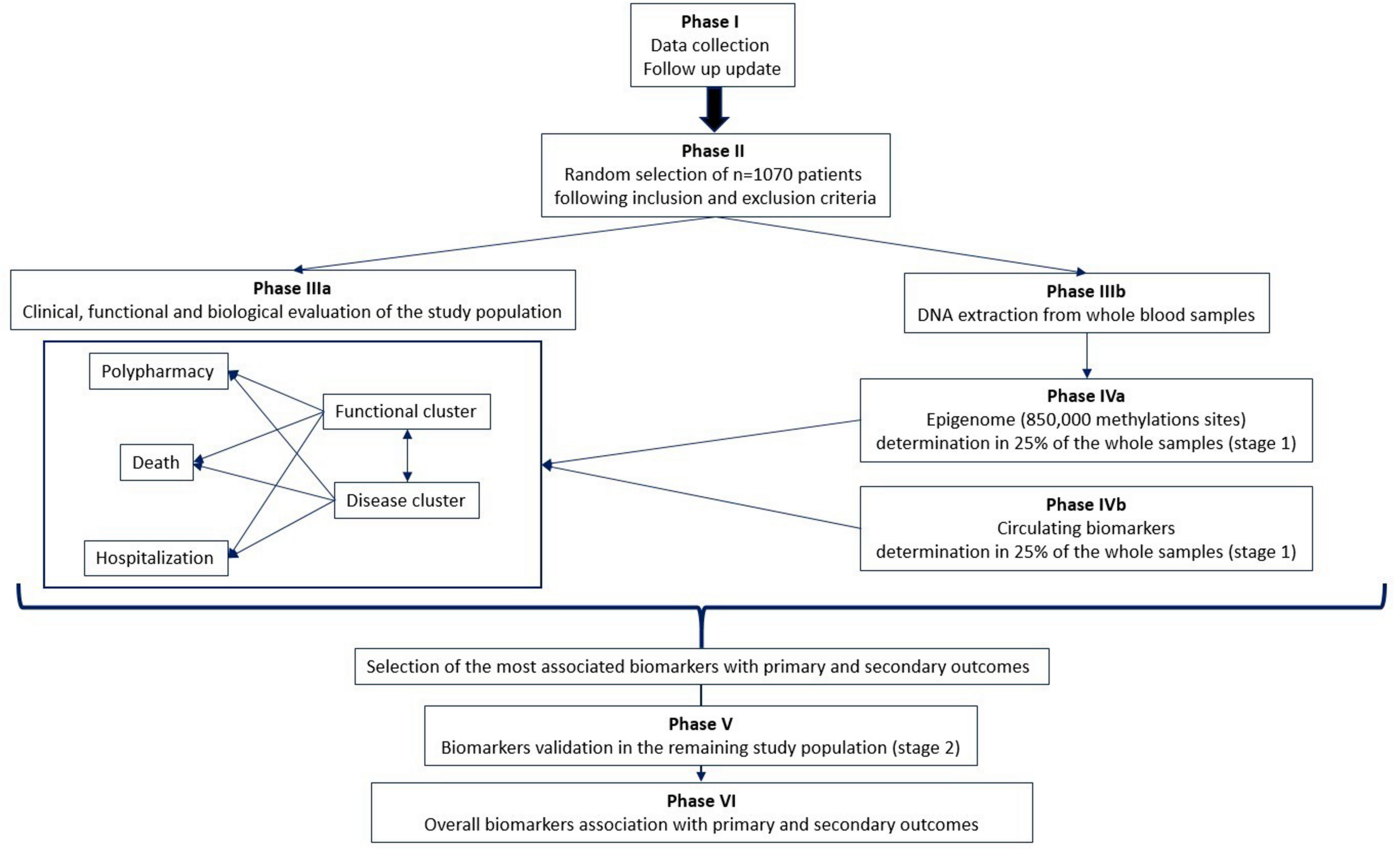
Flowchart of the experimental design.

The project design involves a multi-phase approach (Figure 1).

### Phase I: Follow-up update

Follow-up information on the date of death will be provided for all Reportage project patients for up to 10 years.

### Phase II: Patient selection

One thousand and seventy hospitalized older adults will be randomly selected from the Reportage database on the basis of the following inclusion criteria:

- Age ≥ 65 years.
- Clinical records: two or more of the following conditions on an ongoing basis (present in at least 5% of the overall population): heart attack/myocardial infarction, cerebrovascular disease (CVD), dementia, chronic kidney disease (CKD), hypertension, dyslipidemia, ischemic cardiomyopathy, stroke, PAD, chronic obstructive pulmonary disease (COPD), diabetes, cancer, Parkinson's disease, heart failure, atrial fibrillation, anemia, deafness, hip fracture, thyroid dysfunction, asthma, depression, migraine, eczema/dermatitis, irritable bowel syndrome, osteoporosis, anxiety/panic attacks, rheumatoid arthritis, glaucoma, ear/vestibular disorders, epilepsy, chronic sinusitis, tuberculosis, meningitis, multiple sclerosis, bronchiectasis, prostate problems, schizophrenia, hepatitis, liver failure/cirrhosis, and inflammatory bowel disease.
- Availability of blood and plasma samples at the IRCCS-INRCA: buffy coat and plasma samples (withdrawn at baseline) stored at −80°C.
- Availability of information from baseline to 10 years of follow-up: death, hospitalization, clinical records, functional status, routine laboratory parameters, onset of new comorbidities after the first discharge, and drug intake.

### Phase IIIa: Clinical, functional, and biological evaluation of the study population

Patient selection will be followed by several data mining approaches to evaluate:

- The prognostic weight of MM and functional status in relation to death and hospitalization.
- The relationship between MM, functional status, and polypharmacy in relation to the occurrence of new comorbidities.
- The accuracy of several MM tools, such as the Charlson Comorbidity Index and its derivation, in the prediction of MM patterns, functional impairment, death, hospitalization, and the onset of new comorbidities.

### Phase IIIb: DNA extraction and purification

Following the selection of patients, blood and plasma samples will be collected for the determination of biomarkers. In this phase, DNA is extracted and purified from frozen buffy coat samples for each selected individual.

### Phase IVa: Epigenetics analyses in 25% of the whole sample (stage 1)

Epigenetic analyses on the PROMOTERA project will be divided into two stages: in stage 1, genome-wide methylation profiles, obtained by utilizing the Illumina Infinium MethylationEPIC Array, will be determined as a proportion π_sample_ = 25% (*n* = 272) of the overall PROMOTERA study sample. This population will be selected at random. Stage 1 results will be used to choose a proportion of the *M* CpG sites (Ω_marker_) that are strongly associated with primary and secondary outcomes.

### Phase IVb: Determining aging biomarkers in 25% of the whole sample (stage 1)

In parallel with the epigenetics analyses performed in stage 1, a set of selected circulating biomarkers will be analyzed on plasma samples from the same population. Based on the outcomes of this phase, a subset of these biomarkers will be chosen as a result of their association with primary and secondary outcomes.

### Phase V: Validation of biomarkers in the entire study population (stage 2)

The biomarkers selected in phases IVa and IVb will be validated on the remaining samples (75%, *n* = 798).

### Phase VI: Association of overall biomarkers with primary and secondary outcomes

All the biomarkers validated in stage 2 will be integrated with the clinical, functional and biological data. Several biostatistical and bioinformatics analyses will be conducted to identify biomarker signatures that are strongly associated with primary and secondary outcomes.

The synergy between (a) detailed information about diagnoses for each individual patient, (b) a Comprehensive Geriatric Assessment (CGA), (c) routine laboratory data, (d) epigenetics data, and (e) plasma protein determination will improve the characterization of patients with MM, including the prognostic value for multiple outcomes such as overall mortality, re-hospitalization, and functional decline.

### Outcome variables

#### Overall mortality

Mortality data include the date, place, and causes of death (when available). Up to 10 years' information on survival status (i.e., dead or alive) will be obtained from the Marche Region's administrative database.

#### CGA

CGA was conducted at admission and discharge using the validated interRAI Minimum Dataset for Acute Care (MDS-AC) (51), encompassing identification information, demographic data, functional state assessment, number and type of diagnoses, and number and type of medications, treatments, and procedures (50). Functional status was evaluated on the basis of several dimensions: the number of impaired basic and instrumental activities of daily living (BADL and IADL) (48, 52, 53) as a measure of functional status; the cognitive performance scale (CPS) as a measure of cognitive function; sensory functioning (hearing and vision); history of falls; pressure sores and skin conditions; pain; delirium, and behavioral changes; nutritional status and oral health; urinary incontinence; and, recent weight loss.

#### Use of healthcare resources

Information regarding hospital admissions (number and duration of hospital stays), additional laboratory tests performed, and the use of care services (e.g., nurse home visits, physiotherapy, home help, social transport, and day care facility) were also collected. Up to 10 years of information on the use of healthcare resources will be obtained from the Marche Region's administrative database.

### Exposure variables

#### Demographics

Age, gender, dwelling information, marital status, previous hospital admissions, and family arrangements.

#### Clinical variables

The objective examination includes blood pressure, heart rate, and anthropometric variables (body mass index, BMI). A thorough clinical history was carefully compiled by trained physicians. Diagnoses were coded using the International Classification of Diseases, 10^th^ Edition (ICD10) (54) and the coding of medications was carried out by means of the Anatomical Therapeutical Chemical Classification System (ATC) (55).

### Epigenetic variables

DNA will be extracted from frozen buffy coat samples using standard methods (Ion AmpliSeq DNA Kit). After analyzing DNA quality and quantity with a Nanodrop Spectrophotometer, the Infinium MethylationEPIC BeadChip Kit (Illumina, San Diego, CA, USA) will be used to determine DNA methylation. The Infinium HD Methylation Assay Protocol Guide combines bisulfite conversion of genomic DNA and whole-genome amplification (WGA) with array-based direct capture and scoring of CpG loci.

### Protein variables

Using an immune-affinity assay (ELISA), plasma samples from the study population will be analyzed for selected circulating proteins such as DHEAS, IL-6, CRP, Cyst-C, A2M, and sRAGE.

### Statistical analysis

Baseline characteristics will be described using standard descriptive methods, such as summary statistics and frequency tables. Parametric and non-parametric statistical approaches (such as contingency table analysis, regression, and analysis of variance) will be used when appropriate.

Data pre-processing will be performed using the R package *minfi* (56) and a DNA methylation level will be summarized for each CpG site by calculating a “*beta*” value ranging from 0 to 100%. CpG probes will be treated as missing if the detection *P*-value > 0.01, and CpG sites with more than 5% missing data will be excluded from the analysis.

Epigenetic age will be calculated by multiplying beta values by the regression weights from Horvath [353 CpG sites (35)], Hannum [71 CpG sites (37)], and Levine [513 CpG sites (38)] to create the respective clocks. Delta age (Δage) will be defined as a simple subtraction of the chronological age from the epigenetic age using all the versions of the clock mentioned previously. In all cohorts, cell count predictions will be estimated from DNA methylation data using the Houseman method (57).

The determinants of overall 10-year survival will be calculated using Cox proportional hazards analysis including Δage as independent variables, and corrected for the following confounders: age, gender, clinical, functional, or biological data. The accuracy of exposure variables in predicting mortality will be estimated using Harrell's C-statistics. The same analyses will be repeated by adding the individual age-associated CpGs (one at once) to the Cox model. A two-stage approach will be adopted to validate individual CpG sites associated with mortality. Initially, all of the more than 850,000 methylation sites analyzed by the Illumina Infinium MethylationEPIC Array will be analyzed in a proportion π_sample_=25% of the study sample in stage 1 (*n* = 272), and the results of stage 1 will be used to select a proportion of these *M* CpG sites (Ω_marker_) for follow-up on the remaining sample in stage 2 (75%, n=798). The parameter Ω_marker_ will be selected on the basis of the corresponding *p*-values within the context of the survival analysis.

The validity of the estimated coefficients will be internally verified using a non-parametric bootstrapping resampling analysis with replacements.

In order to reduce the complexity of MM, a dimensionality reduction approach will be performed by clustering patients into a few groups on the basis of the most frequent diagnoses at baseline for each stratum in order to obtain a classification of patients according to an MM pattern. The risk analysis will be repeated for each MM pattern. The association between the epigenetic clock and MM patterns will be determined by means of a Generalized Linear Model. Multiple adjusted models will be developed to consider each possible covariate that could influence the significance of the results and to identify independent variables linked to primary and secondary outcomes. Survival analysis with Kaplan Meier estimates will be performed to assess and compare survival over time among the clusters (Log-Rank test). Cox proportional hazards models will be used to assess and compare survival-time outcomes in each cluster in the presence of possible confounders. Several network medicine-based approaches will be developed in order to build the Human Interactomes and Disease Networks (58). Finally, we will investigate the additive effect of the epigenetic clock on the predictive ability of MM patterns. Changes in Harrell's C and the categorical Net Reclassification Index (NRI) will be calculated with 1,000 bootstrap samples to estimate the 95% CI. A two-tailed *p*-value of < 0.05 will be considered statistically significant. The Bonferroni correction for multiple comparisons will be applied to control for the risk of false-positive results. Statistical analyses will be conducted using the Stata 15.1 Software Package for Windows (Stata Corp, College Station, TX).

### Sample size

The sample size was calculated on the basis of the primary objective (the association between the epigenetic clocks' Δage and mortality). For this reason, we planned to include 1,070 samples of individuals from the Reportage Project. This sample was obtained by setting α = 0.05, β = 0.2, and using a Hazard ratio = 1.23, a 10-year follow-up period, and a median survival time of 6.2 years for patients whose Δage was not expected to increase. This sample size is also in line with other study populations in which the prognostic value of several epigenetic clocks were assessed (36). This sample will enable the detection of significant associations between epigenetic clock Δage and mortality during the 10-year follow-up period.

## Discussion

MM affects 13% of the general population, and its incidence increases with age (59, 60). MM is associated with a lower quality of life, changes in the likelihood of hospital admission, depression, frailty, functional impairment, and polypharmacy (61–63). Several studies have attempted to measure the severity of MM and its relationship to mortality (6–9, 29). However, most of the research that sought to estimate the risk of death in MM patients by using multiple comorbidity indexes indicated a high risk of bias (29).

To date, neither of the MM patterns nor the measure of MM through several mortality indexes have been able to provide an accurate estimate of the severity of MM and the associated risk of adverse health outcomes.

In the era of *omics* and *network medicine*, therefore, characterizing the MM phenotype through the integration of several omics profiles to clinical and functional status could increase knowledge on the pathways involved in MM, thus improving the management of patients and reducing the burden of chronic diseases on healthcare systems.

The molecular signature of patients with MM, along with a 10-year functional and clinical profile that includes drug prescriptions and hospitalizations, will enable us to identify surrogate biomarkers of these features that could potentially be implemented in a clinical setting to identify preventive care strategies and personalized treatments.

Our study seeks to improve the prognostic value of MM in terms of mortality and functional decline throughout a 10-year follow-up period. Moreover, the project strives to characterize MM patterns and assess their relationship with functional impairment. To date, no studies have examined the wide spectrum of relationships between MM patterns, health outcomes, polypharmacy, and several biomarkers associated with healthspan over a 10-year time frame. MM patients are more susceptible to increased risk of disability and hospitalization; therefore, they are not included in clinical trials specifically incorporating biomarkers and functional status.

The availability of a comprehensive longitudinal database in synergy with new biomarkers will also allow us to evaluate how and which inappropriate drug decision-making could lead to the onset of new comorbidities or to functional impairment. Disentangling the relationship between polypharmacy and associated disease outcomes could prevent the onset of new chronic conditions and their relative costs for the National Health System, as well as improve the quality of life of older adults. Providing precise, personalized care is the cornerstone of health services designed for MM patients.

We also aim to construct an accurate, multicomponent algorithm that is inclusive of clinical, functional, and molecular data, capable of predicting at an early stage the onset of new comorbidities, death, and functional decline within 10 years of follow-up. Once validated, this tool could be made available to primary care practitioners for the screening, early diagnosis, and treatment of MM patients, thereby decreasing risks to individuals.

Our study's longitudinal design will also enable us to evaluate the quality of care provided to MM patients and to develop new multidisciplinary personalized care plans based on their clinical, functional, and biological profile. On the basis of the projected risk of adverse health outcomes, tailored, coordinated, and integrated treatment and long-term follow-up strategies will be formulated for each patient. This study will support an integrated health system built around systems medicine and strategic partnerships to manage MM. It could include: (i) an understanding of the social, economic, environmental, epigenetic determinants, molecular, and cellular mechanisms underlying MM; (ii) primary care and practice-based interprofessional collaboration; (iii) carefully phenotyped patients; (iv) the development of unbiased and accurate biomarkers for multiple morbidities, severity, and follow-up of patients; (v) socioeconomic science; (vi) development of guidelines; (vii) training; and (viii) policy decisions.

This approach is expected to improve the implementation of involved personalized medicine in clinical practice to reduce unfavorable outcomes and the deterioration of health conditions in the older population, improve access to healthcare services, and consequently, reduce healthcare expenditure.

The findings of this study are expected to facilitate the transition from conventional medicine into the era of network medicine, where pathway-informed molecular diagnostics will enable the selection of therapy in accordance with the paradigm of precision medicine.

The transition from healthcare provision to personalized medicine requires making new knowledge accessible, placing a greater emphasis on patient perspectives, recognizing the value of molecular pathways in guiding care, building new infrastructure and information management processes, and reshaping healthcare delivery to ensure access to personalized medicine technologies and services (64).

## Strengths and limitations

This study will be able to rely on a comprehensive database containing clinical, functional, and biological information on more than 1,000 MM patients who have been monitored for 10 years. This project will highlight the most significant diagnostic and prognostic clinical, functional, and biological markers of MM, thereby recommending their implementation in clinical settings to identify those patients with a higher risk of death, hospitalization, or functional decline. Moreover, this study will enable the identification of those drug interactions that affect older adults. In parallel with patient involvement and empowerment, this study seeks to contribute to improvements in healthcare policies and their implementation in practice.

This study's additional strengths include: (i) the sample cohort composed of hospitalized patients over 65 years of age. For this reason, we expected to find higher mortality rates than those observed in a community-dwelling population. This should significantly reduce the number of censored observations and, consequently, the required sample size to detect these potential modifiers; (ii) the relatively long follow-up period that will allow us to adjust for several confounders and mitigate several sources of bias; (iii) clinical, functional, biological, and mortality data accuracy acquired over 10 years of monitoring; and iv) a dimensionality reduction approach that will be performed to decrease the number of variables by clustering patients into a few groups based on the MM pattern (i.e., cancer, cardiovascular, and neurological) or the functional status.

One limitation of the study is that the sample size could be smaller than required to detect weak associations between the measured biomarkers and potential modifiers of the association between epigenetic age acceleration and mortality risk during the follow-up period. The sample size was designed on the basis of the primary objective of discovering significant associations between epigenetic clocks' Δage and mortality during the 10-year follow-up period. However, due to the number of potential tested modifiers, the Bonferroni correction for multiple comparisons will be applied to prevent false-positive findings. This study will recommend introducing several biomarkers in clinical settings to improve the prognostic value of MM. However, this translational protocol necessitates several estimation and validation phases. A limitation of the project could be the feasibility of a wide-scale implementation of these biomarkers in clinical practice. Nonetheless, the involvement of the most important stakeholders and companies would make it possible to develop new diagnostic and prognostic experimental procedures.

The absence of information regarding the causes of death of patients who died outside of hospital may also be considered to be another limitation of the study. For this reason, the association analysis on causes of death will only be utilized for descriptive purposes and will not be used in the main analyses in order to eliminate their potential influence on the outcomes.

## Ethics statement

The studies involving human participants were reviewed and approved by Ethical Committee of the Italian National Research Center on Aging (CE INRCA 20031, 04/02/2021). The patients/participants provided their written informed consent to participate in this study.

## Author contributions

FP is the lead investigator of the study and obtained the necessary funds. MD, MF, and FM also contributed to write the project and obtaining the funding. MD and LS will coordinate the statistical analysis. MF will coordinate the clinical evaluation of the results. FM will coordinate the healthcare consumption evaluation and will provide all the information from the administrative database. AM will coordinate the epigenetic evaluation of the results. RL will provide the update of the follow-up information. AB revised the study protocol submitted to ethical committee and will provide the biological samples. SB is responsible of the reportage project and provided all the information already present in the database. ACo, ACh, MP, and FL will provide the overall supervision of the project. All authors have substantially revised the study protocol and approved the submitted version of the paper.

## Funding

This study was supported by Ricerca Finalizzata funding from the Italian Ministry of Health Grant and the Marche Region (Grant Number GR-2019-12368606) to FP and IRCCS INRCA.

## Conflict of interest

The authors declare that the research was conducted in the absence of any commercial or financial relationships that could be construed as a potential conflict of interest.

## Publisher's note

All claims expressed in this article are solely those of the authors and do not necessarily represent those of their affiliated organizations, or those of the publisher, the editors and the reviewers. Any product that may be evaluated in this article, or claim that may be made by its manufacturer, is not guaranteed or endorsed by the publisher.

## Abbreviations

MM: multimorbidity
CGA: comprehensive geriatric assessment
hsCRP: C Reactive Protein
IL6: Interleukin 6
DHEA: Dehydroepiandrosterone sulfate
A2M: Alpha-2-Macroglobulin
sRAGE: Soluble receptor for advanced glycation end product
Cyst-C: cystatin C
ADL: activity daily living
CKD: chronic kidney disease
COPD: chronic obstructive pulmonary disease
BMI: body mass index
ATC: Anatomical and Therapeutical Chemical classification system.
